# Medicines used in mental, neurological and substance use disorders in Gauteng, South Africa: A secondary analysis of the 2017–2018 provincial pharmaceutical database, Part 1

**DOI:** 10.4102/sajpsychiatry.v27i0.1552

**Published:** 2021-01-28

**Authors:** Jade C. Bouwer, Shereen Govender, Lesley J. Robertson

**Affiliations:** 1 Department of Psychiatry, School of Clinical Medicine, University of the Witwatersrand, Johannesburg, South Africa; 2 National Department of Health, Pretoria, South Africa

**Keywords:** essential medicines, prescribing patterns, rational use, psychopharmacology, Anatomical Therapeutic and Chemical classification/Defined Daily Doses

## Abstract

**Background:**

Access to essential medicines is an integral part of effective health systems. Analysis of medicine procurement may assist with ensuring sustainable access.

**Aim:**

To describe the profile and cost of medicines procured for managing mental, neurological and substance use (MNS) disorders during the 2017–2018 financial year.

**Setting:**

The study was conducted in the public health sector in the Gauteng province, South Africa.

**Method:**

A secondary analysis of the Gauteng Medical Stores Administration System database was performed. Medicine procurement for managing MNS disorders was analysed descriptively by using the World Health Organization’s Anatomical Therapeutic Chemical/Defined Daily Dose (ATC/DDD) methodology. Procurement of each medicine was evaluated in local currency (Rands) and in DDD/1000 population served. The District Health Information System was used to estimate population served.

**Results:**

Of the total provincial medicines expenditure in 2017–2018, 3.73% was for MNS disorders, which is similar to the spending on cardiovascular (4%) and respiratory (3%) disorders. Antivirals for systemic use comprised 44% of the total expenditure, followed by vaccines at 13%. Of the medicines for MNS disorders, 32.5% of DDDs procured were for anti-epileptics (ATC N03A) at 47.5% of expenditure; 26.2% of DDDs were for antipsychotics (ATC N05A) at 30.9% of expenditure; and antidepressants accounted for 30.8% of DDDs at 6% of expenditure.

**Conclusion:**

Less than 4% of provincial medicines expenditure was on medicines for MNS disorders, of which almost 78.4% of expenditure was on anti-epileptics and antipsychotics. With limited financial resources, evaluation of procurement patterns raises awareness of relative costs.

## Introduction

Access to essential medicines is one of the World Health Organization’s (WHO) six building blocks for an effective, efficient and equitable healthcare system. Included in sustainable development goal 3.8, essential medicines are integral to achieving universal health coverage.^1^ The WHO describes an essential medicine as one that satisfies a population’s priority healthcare needs,^2^ and it has developed an essential medicine list (EML) to guide low- and middle-income countries (LMICs)^3^ in procurement and supply-chain management. Access to these medicines relies on four important aspects: rational selection, affordability, accessibility and appropriate use.^4^ These factors should be considered prior to granting a medicine EML status.

Selecting psychotropic medicines for an EML according to the best clinical evidence can be difficult.^4,5^ Randomised-controlled trials are often heterogenous or poorly generalisable, and may be subject to sponsor bias. Research in the psychiatric population requires careful navigation around issues of capacity to consent and ethical hurdles in drug trials. Furthermore, many psychotropics have overlapping indications, efficacies and tolerability profiles, rendering true treatment benefit unclear. Study results may differ from clinical experience, resulting in clinicians opting to use non-EML items. However, the costs associated with newer psychotropics may compromise sustainable access if unaffordable, especially in LMICs.^5^ Although medicine accessibility is dependent on efficient supply and geographical proximity of healthcare services to the patient population, medicine procurement is often influenced by prescribing patterns.^6^ Prudent prescribing guided by clinical indication, risk–benefit analysis and treatment guidelines can assist in ensuring rational use of available medicines. The WHO, therefore, recommends that Standard Treatment Guidelines (STGs) are used to ensure rational and cost-effective prescribing of safe and efficacious medicines.^3,7^

### Medicines for mental, neurological and substance use disorders

Mental, neurological and substance use (MNS) disorders (commonly grouped together for their similarities in aetiology, disease progression, treatment approach and outcome) contribute substantially to morbidity and mortality and incur high social and economic costs. Indirect costs (loss of income or productivity, and absenteeism) are estimated to far outweigh the direct costs of mental illness.^8^ In 2002, lost income was estimated at R28.8 billion, compared with roughly R472 million spent on healthcare.^9^ Whilst effective multimodal interventions may be delivered across all health service levels, evidence-based medicine treatment (although not necessarily curative) is essential to facilitate improved care outcomes.^4,9,10^ However, insufficient allocation of resources may hinder mental health coverage.

In an attempt to improve this, the National Mental Health Policy Framework (NMHPF) and Strategic Plan 2013–2020 advocates for accessible, equitable mental health services on par with general health services and for medicines on the EML to be available at all services. Medicine use should be monitored and evaluated towards quality improvement,^9,11^ with mental health promotion and prevention of illness serving as important components of effective healthcare.

### Role of information systems in ensuring essential medicine access

To facilitate internationally comparable drug utilisation research, the WHO has established an Anatomical Therapeutic Chemical (ATC) classification system for all medicines.^12^ This system assigns a unique code to each medicine starting with a specific letter representing the physiological system on which it acts. For comparison of medicine use, a defined daily dose (DDD) of each medicine is used. The DDD, a statistical measure of medicine consumption, defined by the WHO as ‘the assumed average maintenance dose per day for a drug, used for its main indication in adults’,^12^ is a unit or measurement assigned per ATC code and route of administration (e.g. oral or parenteral). Defined daily doses are not a reflection of individual patient prescription but an average dose based on the literature review and consensus agreement. Use of the ATC/DDD system allows for standardisation of medicine groups and doses, allowing monitoring and comparison of medication utilisation.^12^ Therefore, health information systems play a pivotal role in ensuring that health systems operate effectively and efficiently.^6^

In the absence of accurate recording of service delivery, medicine usage, financing and human resources, policymakers may be unable to develop, implement and monitor healthcare systems, promote research and training and allocate budgets effectively. The procurement of medications for the treatment of MNS disorders may serve as an indirect indicator of health service provision and disease burden.^4^

Psychotropic medicine utilisation patterns have been studied, primarily in high-income European countries, where healthcare data are routinely captured.^13^ Database analysis has shown the distribution of patients between service levels, appropriateness and extent of medicine use, disease profiles, resource distribution, total expenditure and medicine consumption. A PubMed search was performed on 11 January 2018 to identify South African research on psychotropic prescribing patterns. The terms ‘psychotropic prescribing’, ‘psychotropic’, ‘drug register’, ‘pharmacy database’ AND ‘mental illness’ AND ‘Africa’ were used, with no restrictions. No public-sector studies of a district or provincial database analysis were found.

### Gauteng province monitoring of medicine procurement

South Africa has its own locally relevant national EML and STGs.^3^ Although there is a national tender process to ensure medicine availability, implementation of the STGs requires provincial procurement of these medicines. The 2012 NMHPF recommended provision of essential medicines and monitoring of treatment response.^9^ Although this is not yet achieved, some systems are in place. In Gauteng, pharmaceutical procurement and expenditure for the public health sector are captured by the Gauteng Medical Supplies Depot in the Medical Supplies Administration System (MEDSAS) database. The MEDSAS database keeps record of all medications ordered and dispensed to facilities procuring medicines from the central depot in Johannesburg.

In the absence of patient-linked data, facility headcounts and patient day equivalents may be used to monitor service utilisation. These data are captured in the District Health Information System (DHIS) database. Although not specific to mental health visits, it provides comparative statistics regarding the population served. Together, these databases may thus provide an indirect indication of the disease burden in receipt of healthcare through the related medicine costs.

This article is the first part of a series of articles from the same study, in which medicines procured during 2017–2018 were analysed in terms of relative proportions of procurement between medicines, service levels and institutions. In this article, we aimed to describe the profile and cost of medicines procured in Gauteng for managing MNS disorders during the 2017–2018 financial year and to place this in the context of total medicine procurement in the province as per the WHO ATC classification.

## Methodology

An exploratory, quantitative analysis of the profile and cost of medicines procured in Gauteng for the 2017–2018 financial year was performed. A secondary analysis of the MEDSAS database was conducted. Descriptive statistics were used to analyse the DDD/1000 population of each of the medicines procured and the cost/1000 served. The study parameters were described according to the Reporting of studies Conducted by using Observational Routinely collected health Data (RECORD) Statement.^14^

### Study setting

The study was conducted in the public health sector of the Gauteng province. According to the 2011 National Census, Gauteng had a total population of 12 272 263.^15^

### Study population

Medicines that act on the nervous system are classified under the letter N in the ATC system. The study population comprised medicines procured for the treatment of MNS disorders in the ATC classes N03–N07 (Table 1). For inclusion in this study, the medicine must be used in the treatment of MNS disorders and procured by Gauteng for the public health sector.

**TABLE 1:** Anatomical Therapeutic Chemical third-level classification and description.^12^

ATC third level	Description
**N03A**	Anti-epileptics
**N04A**	Anticholinergics
**N04B**	Dopaminergics
**N05A**	Antipsychotics
**N05B**	Anxiolytics
**N05C**	Hypnotics and sedatives
**N06A**	Antidepressants
**N06B**	Psychostimulants
**N07B**	Drugs used in addictive disorders

ATC, Anatomical Therapeutic Chemical.

Medicines procured on behalf of contracted care (e.g. Life Healthcare Esidimeni) were excluded, as well as medicines belonging to the N class used for anaesthetic (N01), analgesic (N02) or specialised neurological services. This included specific third-level groupings of medicines related to the above ATC classes, such as N06C (psycholeptics and psychoanaleptics in combination), N06D (anti-dementia drugs), N07A (parasympathomimetics), N07C (antivertigo preparations) and N07X (other nervous system drugs). The DDD for each medicine (not per pack) was obtained from the WHO Collaborating Centre for Drug Statistics Methodology website.^12^ All DDDs in the provincial database (except for lithium carbonate, where the DDD of 166 milligrams (mg) was historically used in the provincial database) correspond to the ATC/DDD index. The EML status of medicines was derived from the National Department of Health STGs.^3^

District Health Information System data were used to establish total headcounts of population served at Gauteng healthcare facilities according to service levels. District Health Information System uses the term ‘headcount’ for district clinics and ‘patient day equivalents’ for inpatient facilities. In this study, the term ‘headcount’ was used for both.

### Data collection

Medical Supplies Administration System database procurement data are captured by staff in the Directorate of Pharmaceutical Service. S.G., who has previous experience in ATC/DDD methodology, collected the raw data from the MEDSAS and DHIS databases for the period of March 2017 to February 2018 by using the vertical lookup function of Microsoft Excel™. This information was arranged according to healthcare facility and district.

J.C.B. and L.J.R then created a new data set. Using Microsoft Excel™, procurement data were organised according to ATC classification, EML status and DDD values. Data extracted from the MEDSAS database for each medicine included the item description, medicine strength, pack size (e.g. packs of 100, 30 or 28 units), unit (e.g. tablet, capsule and vial), cost and quantity procured by Gauteng facilities.

### Data analysis

For each medicine, the procured DDD/1000 population served and cost/1000 population served were calculated.

The following four steps were involved in calculating the DDD/1000 population served:

- The quantity of each medicine per pack was determined by multiplying the size of each pack (no. of tablets/capsules) by the tablet strength for that pack (i.e. tablet strength [mg] × pack size); for example, a pack of 100 tablets of 5 mg haloperidol contains 500 mg of haloperidol.
- The DDD per pack of medicine procured was then calculated by dividing the quantity of medicine in that pack by the DDD value, according to the WHO ATC/DDD index,^7^ except for lithium carbonate. For example, the DDD of oral haloperidol is 8 mg, therefore the DDD for a pack of 100 tablets is 500 mg/8 mg or 62.5 mg. (Since the DDD for lithium carbonate in the WHO ATC/DDD index is in mmol, the DDD of 166 mg used by the Gauteng Department of Health was used instead.)
- Next, the total DDD procured for each medicine was calculated by multiplying the DDD per pack by the number of packs procured of that medicine in the same formulation.
- The total DDD procured per medicine was then divided by the total population served, by using the DHIS data, to obtain the DDD/headcount. This was multiplied by 1000 to give the DDD/1000 population served.

Calculating all medications in terms of their DDD/1000 allowed for all like medications of the same formulation to be added together regardless of the strength or pack size, thus rendering them analysable.

To calculate the cost per 1000 population served, the cost per medicine was first added together for each medicine formulation. This value was then divided by the total population served according to the DHIS data and multiplied by 1000.

A custom program was written by using the Go Programming Language® to process and aggregate the raw data. This allowed for the generation of tables and graphs of the DDD/1000 and cost/1000, in accordance with the study objectives. Descriptive statistics were used to analyse the total DDD, cost/1000 and DDD/1000 per medicine.

An analysis presented in the Gauteng Provincial Pharmacy and Therapeutics Committee (GPPTC) report was used to evaluate the procurement of medicines for MNS disorders in the context of total medicine procurement.^16^ This report is in the public domain on request from the Gauteng Directorate of Pharmaceutical Services and was used to accommodate restrictions in access to data for which permission was granted (see the note under ethical considerations).

### Ethical considerations

Ethical approval for this study was obtained from the Human Research Ethics Committee of the University. Information was removed to ensure blind peer review. Permission to analyse data exclusively pertaining to medicine procurement and expenditure for MNS disorders for publication purposes was granted by the Gauteng Department of Health Research and Epidemiology Directorate (Ethical Clearance Number: M180612).

## Results

### Procurement and expenditure of medicines used for mental, neurological and substance use disorders

**FIGURE 1:**
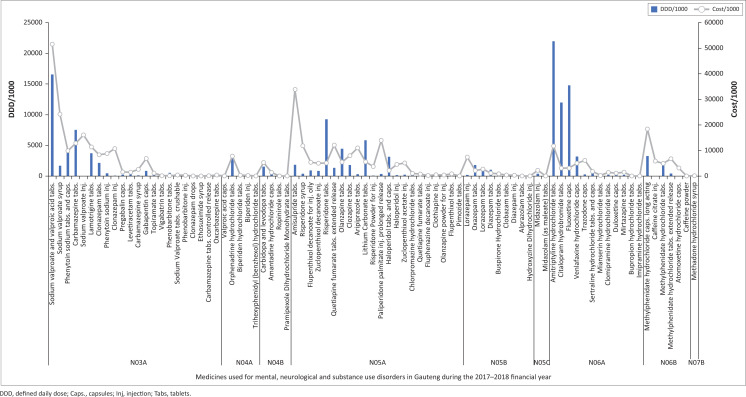
Defined daily dose/1000 and cost/1000 of medicines procured for mental, neurological and substance use disorders in the Gauteng province for 2017–2018.

The total medication procurement amounted to R3 551 613 657.22 in Gauteng province for the 2017–2018 year. Of this, R132 323 280.26 was spent on the study population (those medicines that met the inclusion criteria). This represented 3.73% of the total provincial procurement expenditure. Table 2 shows the summary of the total quantity and cost of medicines procured for MNS disorders according to their ATC classification, with the DDD/1000 and cost/1000 headcount of each of these illustrated in Figure 1. Of the medicines procured, 73.43% of the total expenditure was incurred on EML medicines, 18.65% on non-EML and 7.92% on tertiary/quaternary EML (TQ-EML) medicines (Table 2).

**TABLE 2:** Medicines used for mental, neurological and substance use disorders in Gauteng during the 2017–2018 financial year.^3^

Medicine (ATC fourth level)	EML status	Cost	% Total spend	DDD procured	Cost/DDD
**ATC N03A (Anti-epileptics) in the order of expenditure**					
Sodium valproate and valproic acid tabs.	EML	R24 156 389.00	18.26	7615077.07	R3.17
Sodium valproate syrup	EML	R6 844 078.23	5.17	479800.00	R14.26
Phenytoin sodium tabs. and caps.	EML	R6 540 038.68	4.94	2977266.67	R2.20
Carbamazepine tabs.	EML	R5 739 571.68	4.34	3410840.00	R1.68
Sodium valproate inj.	Non-EML	R5 643 972.05	4.27	14968.27	R377.06
Lamotrigine tabs.	EML	R3 150 386.50	2.38	1011125.00	R3.12
Clonazepam tabs.	EML	R3 048 352.80	2.30	736888.13	R4.14
Phenytoin sodium inj.	EML	R1 908 685.62	1.44	100425.83	R19.01
Clonazepam inj.	EML	R1 881 798.00	1.42	12112.50	R155.36
Pregabalin caps.	Non-EML	R937 089.02	0.71	134484.00	R6.97
Levetiracetam tabs.	Non-EML	R918 934.87	0.69	170995.00	R5.37
Carbamazepine syrup	EML	R760 095.00	0.57	31750.00	R23.94
Gabapentin caps.	Non-EML	R608 706.09	0.46	72705.56	R8.37
Topiramate tabs.	TQ-EML	R310 680.95	0.23	104550.00	R2.97
Vigabatrin tabs.	Non-EML	R126 505.50	0.10	3750.00	R33.73
Phenobarbitone tabs.	EML	R81 967.50	0.06	276722.40	R0.30
Sodium valproate crushable tabs	EML	R48 037.50	0.04	4066.67	R11.81
Phenobarbitone inj.	EML	R43 689.54	0.03	1408.00	R31.03
Clonazepam drops	EML	R25 486.72	0.02	1121.88	R22.72
Ethosuximide syrup	Non-EML	R24 101.22	0.02	624.00	R38.62
Carbamazepine CR tabs.	Non-EML	R22 583.56	0.02	8496.00	R2.66
Oxcarbazepine tabs.	Non-EML	R13 783.00	0.01	750.00	R18.38
Valproic acid caps.	EML	R2072.09	0.00	366.67	R5.65
Subtotal – ATC N03A	-	R62 837 005.12	4.48	17170293.65	R792.52
**ATC N04A (anticholinergics) in the order of expenditure**					
Orphenadrine tabs.	EML	R4 533 386.35	3.43	2045820.00	R2.22
Biperiden tabs.	EML	R134 801.39	0.10	32429.60	R4.16
Biperiden inj.	EML	R62 800.36	0.05	889.50	R70.60
Trihexyphenidyl (Benzhexol) tabs.	Non-EML	R448.29	0.00	40.00	R11.21
Subtotal – ATC N04A	-	R4 731 436.39	3.58	2079179.10	R88.19
**ATC N04B (dopaminergics) in the order of expenditure**					
Carbidopa and levodopa tabs.	EML	R1 514 433.44	1.14	432141.67	R3.50
Amantadine caps.	Non-EML	R905 962.20	0.68	171600.00	R5.28
Ropinirole tabs.	EML	R4335.12	0.00	588.00	R7.37
Pramipexole tabs.	EML	R78 710	0.00	50.00	R15.74
Subtotal – ATC N04B	-	R2 425 517.86	1.82	604379.67	R31.89
**ATC N05A (Antipsychotics) in the order of expenditure**					
Amisulpride tabs.	TQ-EML	R7 167 553.24	5.42	382563.75	R18.74
Risperidone syrup	EML	R5 633 746.75	4.26	167232.00	R33.69
Flupenthixol decanoate oily inj.	EML	R3 624 916.92	2.74	620200.00	R5.84
Zuclopenthixol decanoate oily inj.	EML	R3 142 434.51	2.37	518166.67	R6.06
Risperidone tabs.	EML	R2 805 580.78	2.12	5326764.00	R0.53
Quetiapine ER tabs.	Non-EML	R2 430 210.20	1.84	269512.50	R9.02
Olanzapine tabs.	EML	R2 367 881.43	1.79	1976538.00	R1.20
Clozapine tabs.	EML	R2 193 767.06	1.66	565400.00	R3.88
Aripiprazole tabs.	TQ-EML	R2 120 999.64	1.60	60690.00	R34.95
Lithium carbonate tabs.	EML	R2 015 009.25	1.52	2147364.31	R0.94
Risperidone powder for inj.	Non-EML	R1 736 241.26	1.31	33620.37	R51.64
Paliperidone PR inj.	Non-EML	R1 416 923.04	1.07	31980.00	R44.31
Haloperidol tabs. and caps.	EML	R1 086 513.41	0.82	1205396.25	R0.90
Haloperidol inj.	EML	R1 008 591.53	0.76	28896.25	R34.90
Zuclopenthixol acetate inj.	EML	R98 076.54	0.74	42283.33	R23.13
Chlorpromazine tabs.	EML	R581 588.40	0.44	233060.33	R2.50
Quetiapine tabs.	Non-EML	R238 097.31	0.18	51937.50	R4.58
Fluphenazine inj.	EML	R180 004.86	0.14	178250.00	R1.01
Clothiapine inj.	Non-EML	R78 932.80	0.06	2047.00	R38.56
Olanzapine powder for inj.	Non-EML	R74 962.10	0.06	510.00	R146.98
Flupenthixol tabs.	Non-EML	R29 162.98	0.02	750.00	R38.88
Pimozide tabs.	Non-EML	R5123.11	0.00	212.50	R24.11
Subtotal – N05A	-	R40 916 317.12	30.92	13843374.76	R526.35
**ATC N05B (anxiolytics) in the order of expenditure**					
Lorazepam inj.	EML	R1 588 152.16	1.20	39616.00	R40.09
Oxazepam tabs.	EML	R634 041.11	0.48	490380.00	R1.29
Lorazepam tabs.	EML	R440 633.01	0.33	212520.00	R2.07
Diazepam tabs.	EML	R191 178.00	0.14	167700.00	R1.14
Buspirone tabs.	Non-EML	R189 954.21	0.14	30320.00	R6.26
Clobazam tabs.	Non-EML	R182 146.00	0.14	35000.00	R5.20
Diazepam inj.	EML	R58 005.13	0.04	25160.00	R2.31
Alprazolam tabs.	TQ-EML	R10 088.88	0.01	16200.00	R0.62
Hydroxyzine inj.	Non-EML	R1586.88	0.00	400.00	R3.97
Subtotal – N05B	-	R3 295 785.38	2.48	1017296.00	R62.95
**ATC N05C (hypnotics & sedatives) in the order of expenditure**					
Midazolam inj.	EML	R763 146.22	0.58	123214.33	R6.19
Midazolam tabs.	EML	R896.00	0.00	160.00	R5.60
Subtotal – N05C	-	R764 042.22	0.58	123374.33	R11.79
**ATC N06A (antidepressants) in the order of expenditure**					
Amitriptyline tabs.	EML	R3 255 345.24	2.46	5619584.00	R0.58
Citalopram tabs.	EML	R1 280 575.58	0.97	4972590.00	R0.26
Fluoxetine caps.	EML	R1 026 602.20	0.78	4906548.00	R0.21
Venlafaxine caps.	TQ-EML	R849 670.85	0.64	539347.50	R1.58
Trazodone caps.	Non-EML	R541 320.57	0.41	25333.33	R21.37
Sertraline tabs. and caps.	Non-EML	R267 076.80	0.20	91510.00	R2.92
Mianserin tabs.	Non-EML	R252 890.19	0.19	39970.00	R6.33
Clomipramine tabs.	Non-EML	R230 000.00	0.17	28750.00	R8.00
Duloxetine caps.	Non-EML	R110 242.80	0.08	23002.00	R4.79
Mirtazapine tabs.	Non-EML	R53 858.52	0.04	6240.00	R8.63
Bupropion tabs.	TQ-EML	R20 399.00	0.02	1500.00	R13.60
Imipramine tabs.	Non-EML	R2960.50	0.00	600.00	R4.93
Subtotal – N06A	-	R7890 942.25	5.96	16254974.83	R73.20
**ATC N06B (psychostimulants) in the order of expenditure**					
Methylphenidate LA caps	Non-EML	R5 143 839.43	3.89	916700.00	R5.61
Caffeine citrate inj.	Non-EML	R2 095 579.20	1.58	156.00	R13 433.20
Methylphenidate tabs.	EML	R1 810 446.46	1.37	725090.00	R2.50
Methylphenidate ER tabs.	Non-EML	R243 365.40	0.18	14256.00	R17.07
Atomoxetine caps.	Non-EML	R150 474.60	0.11	1417.50	R106.15
Caffeine powder	Non-EML	R570.00	0.00	2500.00	R0.23
Subtotal – N06B	-	R9 444 275.09	7.13	1660119.50	R13 564.76
**ATC N07B (drugs used in addictive disorders) in the order of expenditure**					
Methadone syrup	EML	R17 958.83	0.01	1009.60	R17.79
**Total**	**-**	**R132 323 280.26**	**-**	**-**	**-**

Caps., capsules; EML, essential medicines list; ER, extended release; Inj, injection; LA, long acting; Tabs, tablets; TQ, tertiary/quaternary; PR, prolonged release; ATC, Anatomical Therapeutic Chemical; DDD, Defined Daily Dose.

Class N03A (anti-epileptic medicines) accounted for almost one-third (32.5%) of the total DDDs procured for the province at nearly half (47.49%) of the total expenditure, amounting to R62 837 005.12. Sodium valproate and valproic acid tablets had the highest total cost (18.26% of total spending). Class N05A (antipsychotic medications and lithium) accounted for 26.2% of the total DDDs procured at 30.9% of total expenditure, whereas class N06A (antidepressants) accounted for 30.8% of all DDDs procured at only 6% of total expenditure.

**FIGURE 2:**
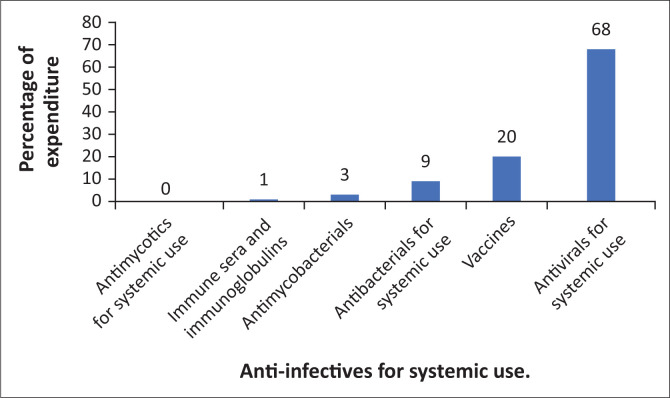
Percentage breakdown of expenditure on anti-infectives in Gauteng province for January 2017–March 2018.

The DDD/1000 population served and cost/1000 population served per medicine are illustrated in Figure 2, with the ATC classes and medicines in the same order as shown in Table 2. Although sodium valproate and valproic acid had the highest cost/1000 headcount served, this was not considerably greater than the DDD/1000 (Figure 2), reflecting high procurement quantities at moderate cost. Amitriptyline (N06A) was the most procured item in DDD/1000. Caffeine citrate (N06B) injection had the highest cost/DDD.

### Expenditure on medicines for mental, neurological and substance use disorders in the context of other disorders

The GPPTC report^16^ published the breakdown of the relative proportion of medicine expenditure per ATC class in Gauteng from January 2017 to March 2018 (Table 3). Although this represents a longer period than the study period, it should not affect the relative proportion of expenditure. According to the GPPTC report, 6% of the total health expenditure was spent on ATC Class N, comprising all nervous system-related medications, including those medicines that were excluded from this study.

**TABLE 3:** Comparison of spending on the different Anatomical Therapeutic Chemical classes as a percentage of total provincial spending in Gauteng, January 2017–March 2018.^16^

Code	Contents	Percentage spend (%)
A	Alimentary tract and metabolism	5
B	Blood and blood-forming organs	7
C	Cardiovascular system	4
D	Dermatologicals	2
G	Genito-urinary system and sex hormones	1
H	Systemic hormonal preparations, excluding sex hormones and insulin	1
J	Anti-infectives for systemic use Antivirals for systemic use	65 68
L	Antineoplastic and immunomodulating agents	3
M	Musculoskeletal system	1
N	Nervous system	6
P	Anti-parasitic products, insecticides and repellents	0
R	Respiratory system	3
S	Sensory organs	1
V	Various	1

The bulk of expenditure (65%) was on anti-infectives for systemic use (Figure 2). Of this, 13% was spent on vaccines and 68% (i.e. 44% of total expenditure) spent on systemic antiviral agents, which includes medicines for the treatment of human immunodeficiency virus (HIV) infectionn. Whilst spending on HIV should not be compromised, the GPPTC report focusses on efforts to rationalise antimicrobial prescribing, which accounted for 9% of spending on systemic anti-infectives. When considered as a percentage of the total spending over the 15-month period, expenditure on antimicrobials equated to 5.85%, similar to the combined spending on anaesthetic, pain, neurological and psychiatric medications (i.e. similar to spending on the entire N class of medicines).

## Discussion

### Procurement and expenditure of medicines used for mental, neurological and substance use disorders

The study revealed that R132 323 280.26 was spent by the Gauteng Department of Health on medicines procured for treating MNS disorders. This accounted for 3.73% of the total Gauteng public health sector pharmaceutical expenditure for 2017–2018. Table 3 shows that 6% of the total budget was spent on nervous system medications over the 15-month period. It would be reasonable to assume that roughly 2.3% was spent on anaesthetic, analgesic and specialised neurology medicines.

Information, such as mental health coverage and treatment outcomes, would be needed to accurately comment on whether spending 3.73% of the pharmaceutical budget is appropriate. Although most LMICs spend less than 1% – 2% of their national budgets on mental healthcare,^10^ South Africa appears to be spending more.

Gauteng spent 6.2% of its total 2016–2017 health expenditure on mental healthcare services (excluding non-governmental organisation subsidies), the second highest provincial MHC spending.^10^ Although 6.2% of the total provincial health expenditure and 3.73% of the medicine expenditure are not directly comparable, it suggests that the bulk of expenditure is on inpatient care and personnel costs, including allied health professionals.

Table 2 shows that almost half of the total expenditure was on anti-epileptic medicines (N03A), followed by spending on antipsychotics (N05A). This is indicative of coverage of more severely disruptive illnesses such as epilepsy, bipolar disorder and psychosis.

Of the total medicines used for MNS disorders, the highest expenditure spent on a single item was for sodium valproate and valproic acid tablets (R24 156 389.00), and this medicine was the most procured total DDD item (Table 2). Despite its high cost/1000 (R51 536.51/1000), it appears that high coverage was attained (high DDD/1000) (Figure 1). As sodium valproate is on the EML at all service levels, including that prescribed for epilepsy by nurses in primary healthcare centres (PHC) and for bipolar disorder by doctors, the high procurement therefore suggests a high burden of MNS disorders because of these conditions, notwithstanding a large treatment gap.

As a group, antidepressants were the most procured medicines in DDD/1000. This is in accordance with the high prevalence of anxiety and depression in South Africa.^10^ Amitriptyline hydrochloride tablets were the most procured medicines in terms of total quantity and DDD/1000 (21939.85/1000; the DDD/1000 gives an indication of the proportion of the total headcount being treated with a certain medication). The multiple indications for amitriptyline may limit its validity in inferring the prevalence of depressive disorders by using its high DDD/1000 (21939.85/1000 population, the second highest DDD/1000 procured overall) as a reference value. Although it is more efficacious than fluoxetine and citalopram for the treatment of depression,^10^ its side-effect profile renders it less tolerable and poses a risk of toxicity in overdose, thereby likely restricting its use as an antidepressant. Fluoxetine and citalopram have similar costs/DDD and are preferred agents of choice, recommended by STGs. It is unclear from this study whether the high DDD/1000 of amitriptyline procured was because of large dose prescriptions or greater coverage.

The highest cost/DDD was for caffeine citrate injection (R13 433.20). This is explained by its high unit cost (probably linked to its non-EML status) but relatively small DDD procured (and, therefore, DDD/1000). This result may be spurious as caffeine citrate is not in the WHO ATC list, and so the DDD used in the MEDSAS database was that of caffeine. Although it is classified as a psychostimulant (N06BC01), it is not in the EML for mental health disorders. Despite its potential use in the treatment of migraine headaches and in the management of preterm babies, further evaluation of its procurement is warranted considering its cost.^10^

This study provides valuable insights into procurement and expenditure patterns in mental healthcare in Gauteng. Comparative studies of procurement and expenditure could promote conscientious prescribing by flagging irregular use and high costs and inspire further investigation to establish appropriateness of medicine use and identify novel outcomes. Knowledge of total medication expenditure may allow for more informed budget allocation, whereas insight into coverage and service provision by facility may assist in guiding resource allocation. However, several factors limited the results. The absence of patient-level data, describing treatment indications and dosages, limits the possibility of drawing inferences regarding high cost or procured items. As a result, many of the observations made in this study are anecdotal. Defined daily doses may not represent the actual clinical usage and local prescribing patterns.^17^ They cannot, therefore, be used to reflect the prevalence of disease accurately but may suggest the extent of disease burden or treatment coverage. Cost/1000 cannot be used as an indicator of disease burden, as high cost/1000 could elude to more expensive medications being used, rather than greater quantities. Neither of these parameters should be assessed in isolation, but should rather be considered together with cost/DDD, which could give an indication as to whether there is greater coverage of medicines, resulting in higher DDD/1000 or more expensive medications being utilised with greater cost/1000.

The overlap of medicine indications and questions around the accuracy of medicine classification within the ATC N group complicate the potential for procurement patterns to act as markers for disease prevalence (e.g. lithium carbonate under N05B). Also, some DDDs do not necessarily correspond to STGs in SA. These factors may lead to an over- or under-representation of the prevalence of some disorders. As procurement does not equate to treatment adherence, the findings do not reflect actual treatment, but only an indirect estimate of utilisation. The purchasing of over-the-counter medications or subscription to traditional healing practices could also not be accounted for in this study. Data capturing of headcounts at facilities may have been inaccurate, leading to the skewing of data. Additionally, at general facilities, headcounts are not specific to individuals seeking mental healthcare. Assessing mental health headcounts in comparison with medication use could prove more valuable.

### Expenditure on medicines for mental, neurological and substance use disorders in the context of other disorders

When compared with other classes, the proportion of spending on MNS disorders is similar to that of alimentary tract and metabolism (5%), cardiovascular (4%) and respiratory (3%) disorders. Spending on musculoskeletal conditions appears to be low (1%) but may be accounted for by the inclusion of analgesics in the N group.

The enormous burden of disease because of acquired immune deficiency syndrome (AIDS) is apparent in the high proportion of spending on antivirals for systemic use (44%). Studies on global burden of disease showed that HIV was solely responsible for the most years of life lived with disability, disability-adjusted life years and years of life lost for South Africa in 2015.^18,19,20^ Therefore, whilst the need to address HIV as aggressively as possible may preclude increased spending on mental healthcare coverage, the complex relationship between HIV and MNS disorders should be considered.^11^ Mental illness and HIV are highly comorbid and mutually reinforcing.^21,22,23^ Up to 35% and 21% of patients with HIV are thought to have comorbid depression and anxiety, respectively.^21^ Regarding depression, treatment improves the depressive symptoms and may improve anti-retroviral adherence and reduce HIV transmission.^24,25^ Treatment adherence and lifestyle outcomes are also worse in HIV-infected individuals.^26^ In China, a population survey by Chen et al.^22^ revealed a 3.6-fold increased risk of contracting HIV and a 2.3-fold increased risk of syphilis amongst people with bipolar disorder compared with the general population. This implies that there may be a benefit of addressing bipolar disorder in preventing sexually transmitted infections.

Considering an estimated treatment gap of 75% identified by the South African Stress and Health (SASH) study,^27^ and the more recent estimated treatment gap of 91% in Docrat et al.’s cost analysis,^28^ it is likely that increased procurement is required to improve mental healthcare coverage. However, spending on HIV, as well as competing disease priorities such as cardiovascular and respiratory conditions, limits the available budget for managing MNS disorders. It is, therefore, imperative to reduce unnecessary expenditure as pharmacotherapy remains the mainstay of treatment.^10^ For example, the sole indication for the use of anticholinergics in mental healthcare is antipsychotic-induced side effects. Rather than adding an anticholinergic agent to treat these effects, alternative options such as reducing doses when side effects emerge or switching to an agent with less propensity for side effects may be considered, and routine prescribing of anticholinergics with first-generation or intramuscular antipsychotics is discouraged. A clear indication for the use of caffeine citrate, which would justify its high cost/DDD, should also be established. Nevertheless, whilst rational prescribing should be a standard practice, it is unlikely that large savings will be achieved solely through this undertaking, and therefore other strategies will be needed if mental health coverage is to improve.

## Conclusion

Despite the significant burden of MNS disorders, spending on pharmacological management was less than 4%. The large treatment gap in South Africa implies that a much greater amount is needed for procurement of these medicines. The high prevalence of HIV in our setting poses a considerable treatment burden and places extensive constraints on healthcare budgets, limiting spending on other disorders. Evaluation of healthcare should be directed away from cost analysis of individual medicines towards improved health outcomes.

Improved access to care and essential medicines, better service delivery, sound leadership and a strengthened health workforce practising frugal prescribing habits may not only improve health coverage but also reduce the indirect costs of untreated mental illness.

## Recommendations

- Ideally, a patient-linked registry would assist in evaluating rational use of medicines, burden of disease and healthcare costs. Until such a registry can be established, procurement patterns could be compared with treatment indications and outcomes to ensure judicial use of medicines.
- Regular database evaluation is recommended to compare procurement patterns and spotlight irrational use. This may also assist policymakers in establishing STGs and allocating budgets.
- Auditing should be undertaken to ensure judicial use of affordable medicines with beneficial treatment outcomes in accordance with current STGs.
- Monitoring of compliance to STGs may flag consistent use of non-EML items. This may indicate a need to explore its evidence-based outcome for possible inclusion on the national EML.
- Some updating of the ATC classification system may be indicated; for example, lithium could be placed with N03A medicines, which could be considered as ‘anti-epileptics and mood stabilisers’.
